# A Multimodal, Adjustable Sensitivity, Digital 3-Axis Skin Sensor Module

**DOI:** 10.3390/s20113128

**Published:** 2020-06-01

**Authors:** Alexis Carlos Holgado, Tito Pradhono Tomo, Sophon Somlor, Shigeki Sugano

**Affiliations:** Department of Modern Mechanical Engineering, School of Creative Science and Engineering, Waseda University, 3-4-1 Okubo, Shinjuku-ku, Tokyo 169-8555, Japan; tito@toki.waseda.jp (T.P.T.); s.somlor@aoni.waseda.jp (S.S.); sugano@waseda.jp (S.S.)

**Keywords:** tactile, skin, sensor, magnetic, capacitive, multimodal, adjustable sensitivity

## Abstract

This paper presents major improvements to a multimodal, adjustable sensitivity skin sensor module. It employs a geomagnetic 3-axis Hall effect sensor to measure changes in the position of a magnetic field generated by an electromagnet. The electromagnet is mounted on a flexible material, and different current values can be supplied to it, enabling adjustments to the sensitivity of the sensor during operation. Capacitive sensing has been added in this iteration of the module, with two sensing modalities: “pre-touch” detection with proximity sensing and normal force capacitive sensing. The sensor has been designed to be interconnected with other sensor modules to be able to cover large surfaces of a robot with normal and shear force sensing and object proximity detection. Furthermore, this paper introduces important size reductions of the previous sensor design, calibration results, and further analysis of other sensor characteristics.

## 1. Introduction

Tactile sensing provides important information for robots to safely interact with people and the environment [1]. During manipulation, direct tactile information from normal and shear contact force measurements is critically important [2]. Covering large areas of robots with skin sensors is also beneficial for safety purposes, in order to allow robots to appropriately react to external interactions and sense their surroundings [3]. Furthermore, a compliant skin is preferred over a rigid interface to minimize damage from unforeseen collisions and unwanted interactions. Generally, sensitivity and range characteristics for sensors are set during the design stage and cannot be changed after manufacturing or result from a static calibration. This poses a potential limitation to interactions during intricate or high-force demanding tasks because usually, high sensitivity will be preferable for delicate interactions, while a broader range is more useful with larger force values.

In order to address most of these difficulties, the novelty elements of our sensor consist of the ability to measure 3-axis forces, with the possibility to adjust the sensitivity during operation, and the ability to detect an approaching object through capacitive sensing. The modular design of the sensor allows for interconnection between modules to cover large surfaces. Furthermore, the network of sensors can be entirely covered with a soft material without hindering the sensing modalities and to add compliance to the robot.

Our sensor is based on the Hall effect magnetic principle and can measure 3-axis forces with digital output and sensitivity that can be adjusted during operation. In this iteration, we present results verifying that proximity of conductive materials can be sensed—via capacitive sensing—before they interact with the sensor. Moreover, we verify that capacitive sensing could also be used to provide normal force measurements for ranges that the magnetic-based sensor cannot reach. The module is designed with three programmable communication ports to be interconnected with other sensor modules. In this case, the mesh of sensors can cover larger areas of contact for robots and supply 3D tactile and proximity sensing capabilities. Another advantage of the sensor module is that it can be covered with a soft and continuous material (e.g., rubber cover), with no need for discontinuities like gaps or grooves. This increases the robustness of the system, making it more suitable for unstructured industrial environments.

The previous iteration of the sensor, presented in [4], exposed several problematic aspects of the design of the sensor. In this paper we present major improvements on the sensor module that focus on the following four areas:

1. Sensor footprint reduction.
2. Minimizing crosstalk between measuring axes.
3. Calibration and sensor characterization.
4. Multimodal capabilities.

The new sensor prototype presented in this work aims to cover these topics with new ideas and experimentation.

The rest of the paper is divided as follows: Section 2 presents related works. In Section 3, information on the layers of the sensor module and the general operating principle is briefly introduced. Section 4, Section 5 and Section 6 describe modifications to the sensor design and experiments to address the first three items presented above, on which this letter intends to focus. The fourth and last item is addressed in Section 7, where experiment results are presented to test the new modalities of the sensor: capacitive proximity sensing and capacitive normal force sensing. Finally, Section 8 offers conclusions and future works.

## 2. Related Works

Many approaches have been taken to solve the problem of tactile measurements in robotics. The majority of these focus on dexterous manipulation and object handling, and therefore, on tactile sensors embedded into end-effectors—parallel grippers, robot hands, etc. We will refer to this group as manipulation tactile sensors (MTS). Alternatively, a smaller number of researchers focused their work on expanded tactile sensing for larger surfaces of robots (e.g., covering an entire robot arm or body). We will refer to this group as extended tactile sensors (ETS). Clearly, MTS and ETS differ in preferred characteristics. MTS tends to focus on small-sized sensors, high spatial resolution, fast response time, and less frequently, 3-axis force sensing. A general overview of robotic hands and their tactile technology can be found in [5,6].

Humans can handle objects dexterously and robustly, by sensing both the magnitude and direction of each contact force—among other measurements, such as visual and auditive feedback. Sensing shear force is particularly beneficial for slippage detection [7,8]. When compared to a single axis sensor, a 3-axis tactile sensor provides more complete data of the interactions between the sensor and the environment [2]. For example, the work presented in [9] shows that the object recognition rate is higher when using data from 3-axis tactile sensors. Hall-effect-based tactile sensors have been developed in [10,11]. The work of [10] presents a single axis tactile sensor for a robot fingertip. A small, soft, 3-axis tactile sensor called uSkin is characterized in detail in [11]. Both works offer good examples of small-sized sensors of the MTS group that are fast and easy to produce and control. While compact and sensitive, neither solution includes proximity sensing features or a different sensing modality, other than contact force.

Meanwhile, the ETS group would benefit from a wider range of operating forces to cover a larger number of interactions, such as enhanced network connection capabilities and multimodality sensing (e.g., proximity sensing, to try to avoid collisions). Simpler and cheaper manufacturing methods would also be an advantage as they would allow production in large quantities. Some methods of construction and applications of robot skin can be found in the Ph.D. dissertation [12] and the works of [13].

In general, the sensitivity and range of measurement of a sensor are decided to suit a certain task before the sensor is made. After this manufacturing phase, the two characteristics are difficult or impossible to change. A sensor in [14] can have its sensitivity adjusted through a mechanism that modifies the properties of the sensor. However, such a mechanism increases the overall size of the sensor, rendering it unsuitable for certain applications.

Sensors based on capacitive technology can present force or proximity sensing modalities. The sensors developed in [15,16,17] focus on force sensing. In [15], the sensor can measure twelve independent points of normal force on the fingertip of the iCub robot. The sensor in [16] uses four tilted transducers to measure 3-axis force with a total size of 32×25×7 mm. However, it offers only one sensing modality, and the calibration procedure is complex due to the non-linear behavior of the solid silicone block wherein the sensors are embedded. In [17], the authors developed a flexible tactile sensor array based on a polydimethylsiloxane polymer. They present 64 taxels with 3-axis sensing capabilities on an area of 18×18 mm. However, the range of measurement of each taxel is narrow: 0–20 mN of force.

Proximity sensors embedded in grippers with similar characteristics are presented in [18,19,20]. They employ either infrared light or time-of-flight sensors inside a transparent silicone cover. This arrangement is capable of sensing proximity, touch, and force modalities with a single sensor type. However, they all present challenges in calibration—due to differences in lighting conditions and reflectivity indexes of objects—and they require voluminous electronics. Most importantly, the elastomer needs to be completely transparent, maintain its deformation characteristics, and remain clean throughout the life of the sensor, which is not realistic in industrial scenarios.

Researchers in [21,22] present capacitive proximity and force sensing modules. The sensors seem to be designed as ETS to work as robotic skin, using modes of self-capacitance sensing for proximity and mutual-capacitance for normal force measurement. The number of pads used for proximity sensing can be adjusted online, providing flexibility to the module. Although both sensing capabilities can be performed with a single type of sensor, only normal force measurements can be obtained. No information on the isolating foam material is disclosed. Moreover, the overall size of the sensor module (40×40×10 mm), with the supplementary electronic components to process the signals, might result in an impractical solution for larger networks. Our sensor uses the two sensing modalities as well, but on a more compact package, with signal processing in the same module and additional magnetic force, accelerometer, and gyroscope data.

The works of [23] introduce an interesting novel idea of a 3-axis force sensor consisting of two layers of force-sensitive resistor arrays separated by a dielectric material. The sensing elements can be placed in a flexible substrate and are small enough to fit 16 units in an area of 5×5 mm. Normal and shear force experiments are presented to prove the concept of the sensor, but no calibration or other characteristics—e.g., the range of operation—are discussed in detail. Furthermore, the manufacturing processes of the sensor and controlling electronics are complex and time-consuming, respectively, and post-processing image recognition is necessary to detect shear forces. No possibilities of expansion into networks to cover larger surfaces are suggested.

Only a few works focus on large-scale robotic skin. The work of [24] presents the iCub robot skin, a capacitive-based array of sensors with good adaptability to curved surfaces and mature design and electronics (commercial product). However, the skin offers only the single modality of normal force sensing. The works presented in [25,26,27] are likely the most advanced and developed robot skin up to date. Their solution consists of sensor modules that can be interconnected to cover large surfaces. In [26], several challenges of a large-scale robot skin are mentioned and an impressive number of 1260 skin cells are interconnected and tested. Furthermore, each module presents multi-sensing modalities of force, proximity, and temperature, and well-developed and thoroughly studied communication protocols. The ability to sense more than one modality is valuable because more available data can provide a better understanding of the surroundings and the situation of a robot, which in turn would offer more effective ways to interact with the environment. Despite the accomplished multi-modality and interconnection features of these sensors, they present two important limitations: only normal force interactions can be measured and a small aperture for the optical-based proximity sensing is required, which means the network cannot be covered. Therefore, the entire surface of the skin might not be compliant.

Our approach is a similar idea, developed for the ETS group, and presents the following features: adjustable sensitivity based on magnetic force sensing and multimodal capacitive sensing; namely, proximity and normal force sensing. Moreover, it can be completely covered with a flexible material providing increased compliance and robustness. To the best of the authors’ knowledge, no other implementation of a robotic skin module has these features combined in a compact package.

At the current stage, the cost of producing a single prototype is between 20 and 25 USD, with the most expensive component being the BMF055 chip (close to 15 USD, for low quantity orders). However, we suggest that the cost could be decreased with minor design adjustments to make the module suitable for production in large batches.

## 3. Working Principle of Sensor

The sensor works using the Hall effect, relying on measuring the change of position of an induced magnetic field over time. Its working principle can be summarized as follows: an electromagnet is placed on top of a flexible material, which is placed on top of a geomagnetic sensor. When external forces are exerted on the electromagnet, the flexible material deforms and the magnetic field moves. Finally, the change in position of the magnetic field is measured by the 3-axis geomagnetic sensor and outputted in digital format.

For the sensor presented in the current paper, we have added two supplementary sensing modalities based on capacitive sensing: proximity sensing and normal force sensing. By adding a conductive layer and a capacitive sensor circuit, the sensor can perceive the approach of conductive objects, thereby providing "pre-touch" capabilities to the module. Employing the same components, additional normal force data are provided by the capacitive circuit.

**Figure 1:**
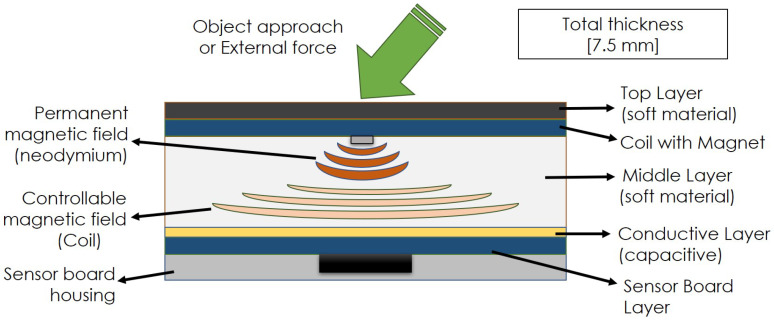
Layers of sensor module.

The sensor module is a layered structure (see Figure 1) where each layer serves a specific purpose. From bottom to top, details of each layer:

- Sensor board layer: The rigid base layer of the module, hereafter “sensor board”, contains all sensing electronics.
- Capacitive layer: A conductive thin layer connected to a capacitive sensing circuit, to sense the proximity of approaching conductive objects and output normal force data (two different modalities).
- Middle material layer: A soft, deformable material placed between the sensor board and the coil board.
- Coil board Layer: The top layer of the sensor, hereafter “coil board”, is mounted on top of the middle layer. This layer is in contact with external physical forces.

The sensor board is a custom printed circuit board (PCB) that includes a BMF055 chip and all necessary electronic components to operate it. Inside the BMF055 there is an ATSAMD20J18 microcontroller and a 9-axis inertial measurement unit: an accelerometer (BMA280), a gyroscope (BMG160), and a geomagnetic sensor (BMM150). All manufactured by Bosch. The geomagnetic sensor inside the BMF055 chip measures position changes in the magnetic field generated in the top layer of the sensor. Furthermore, having processing capabilities on each of the skin modules is favorable because basic signal processing can be done in situ without encumbering the communication bus or the main processing unit of the skin. Additionally, in this iteration, capacitive measurements are introduced. These measurements are now handled by a separate circuit, but they could also be tunneled through the microcontroller. More research on this feature will be conducted in the future.

Importantly, in this iteration it was decided to mount the sensor board upside down. This mounting position adds robustness to the design of the sensor, protecting it from destructive external forces and providing a flat surface on which to place the next layers. The orientation change requires a correction in two of the magnetometer measuring axes (inverting their values) but does not otherwise interfere with the principles of operation of the sensor.

On top of the surface presented by the sensor board, we place a conductive tape that connects to a capacitive sensing circuit. More information on the circuit and features of the new sensing modalities for the sensor can be found later, in Section 7.

A layer of flexible material is placed on top of the conductive tape. In past works [28], we have found that neoprene of 5 mm in thickness works well for both normal and shear force measurements, so we adopted the same material for the current iteration.

Finally, the coil board is glued on top of the flexible material. The coil board consists of an electromagnetic coil and a permanent magnet in the middle. The magnetic field strength on the coil can be adjusted with the current that passes through it. Due to the in-built characteristics of the geomagnetic sensor, where the operating range of *Z*-axis is wider than that of *X* and *Y* axes, the permanent magnet in the middle of the coil provides an increase in magnetic field density towards the center of the coil, thereby using a wider range in *Z*-axis measurements. These results were presented in [4].

During the redesign process to reduce the size of the sensor, the shape of the middle material and the coil board had to be revised and modified. The process is described in the following section.

## 4. Sensor Footprint Reduction

The size of the sensor module has been a matter of discussion since the first version was presented in [29]. A smaller sensor could be used in a higher spatial density mesh structure, providing higher-detail contact information. Therefore, in this work we focused on reducing the size of the sensor.

The main factor influencing the size of the sensor module has been the coil board, which measured 30.5×30.5 mm. Modifying the coil characteristics regarding size, thickness of the copper traces, or another aspect of its morphology directly impacts on the amperage value that can be used on the coil. In turn, this is directly related to the generated magnetic field strength and shape. These changes then influence the required characteristics of the deformable middle material that is placed in between the sensor board and coil. Furthermore, the power consumption of the sensor is closely related to the power consumption of the coil board. Previous versions of the PCB coil [4,28] needed direct current values of as high as 1500 mA, making the temperature of the PCB an important point to consider. Specifically, in [28], several current values were tested and a subset was adopted to keep the temperature of the coil below $45{\;}^{\circ }$C.

### 4.1. New Coil

In order to isolate the effects of the redesign of the coil as much as possible, and to make past results valid, a coil with similar magnetic strength is desirable. Therefore, the same permanent magnet presented in [4] was used to augment the magnetic field towards the center of the coil, and the 5 mm of separation between the sensor and coil was maintained.

**Figure 2:**
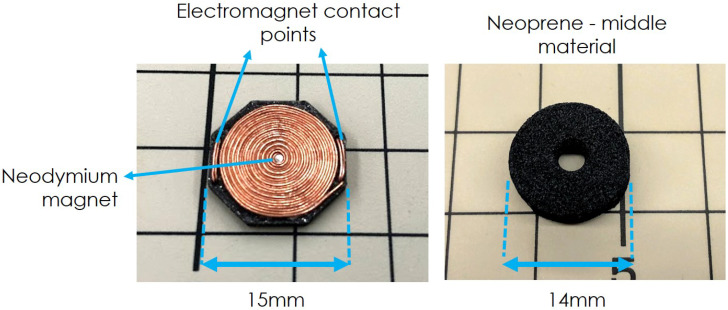
Left: New coil. Manually wound, using enameled copper wire 0.26 mm in diameter. A small neodymium magnet is pasted in the center of the coil. Right: neoprene disc of 14 mm of diameter with a hole in the middle.

Consequently, a new coil design is proposed and presented. The new coil is assembled on a hexagon-shaped 3D printed plastic substrate of 15 mm by side and 0.8 mm in thickness. Enameled copper wire of 0.26 mm in diameter was manually wound in a spiral to produce a flat coil over the substrate. Figure 2 shows a manufactured coil. The new coil is 1/4 of the area of the previously used PCB embedded coil [28].

Based on the American wire gauge (AWG) standard, the diameter of the copper wire used for the coil is very close to the AWG30 gauge. The rated current value for this gauge is 0.52 A for operation under $60{\;}^{\circ }$C. Then, a maximum current value of 500 mA was selected to be tested with the new coil.

Two experiments were performed on the new coil with the selected maximum current of 500 mA passing through it. On the first one, we checked the temperature of the coil. The second piece of experiment checked the generated magnetic field strength and compared it with the previous version of the embedded PCB coil.

For the temperature experiment, values were captured using a thermographic camera from FLIR (FLIR One Pro). Temperature readings were taken every 30 s; however, after 10 min of operation in the open air, the temperature remained constantly under $40{\;}^{\circ }$C, considered suitable for our needs. Results show that the temperature of the coil does not surpass the normal commercial temperature ratings for circuits (between $0{\;}^{\circ }$C and $70{\;}^{\circ }$C), even at the maximum current value of 500 mA. More importantly, the current value of 500 mA has added benefits as it corresponds to 1/3 of the previously employed maximum current of 1500 mA, significantly decreasing the power consumption of the coil. Quantification of reduced power consumption will be researched in future work.

For the generated magnetic field strength experiment, the coil was mounted on a manual XYZ table (from Proxxon, Wecker, Luxemburg) facing upwards. The maximum design amperage value of 500 mA was applied to the coil, and magnetic field strength values were acquired using a magnetometer (MG-3003SD from Lutron, Coopersburg, PA, USA). The magnetometer was fixed on the *Z*-axis of the table, and then the table was manually operated, taking 1 mm steps on the vertical axis away from the sensor. Fifteen values were recorded with the magnetometer and averaged to be assigned to each distance point.

**Figure 3:**
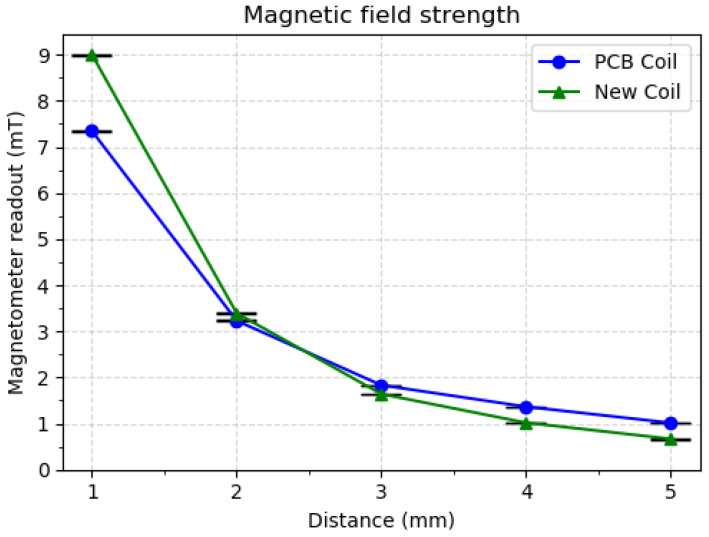
Magnetic field strength results. Values from the previous PCB coil and new coil present similar characteristics.

Results for the magnetic field strength experiment can be found in Figure 3. Values for the “new coil” and the previous coil (“PCB coil”) are presented on the same plot.

These results verify that comparable magnetic field strengths can be achieved with the new coil, even though its size has been reduced to 1/4 of the previous iteration.

As can be seen from our results, the properties of the new coil fit our requirements, and it was therefore adopted as a replacement for the previous coil PCB of the sensor module.

A related point to consider is that the magnetic sensor BMM150 included in the system on chip (SoC) BMF055 is a geomagnetic sensor. As such, it is capable of sensing the low-intensity values of the Earth’s magnetic field. These values depend on location and fluctuate between 24μT and 65μT [30]. We argue that this range does not consume too much of the sensing spectrum, which is 2500μT for the *Z*-axis and 1300μT for *X* and *Y* axes. Furthermore, the influence of the magnetic field of the Earth can be compensated by taking into consideration the absolute orientation of the sensor. As mentioned in Section 3, the BMF055 includes 3-axis high-definition accelerometer and gyroscope on the same SoC, so compensation can be performed directly by the chip without encumbering the master controller.

### 4.2. Middle Layer Material

The reduction in the size of the coil determines that the next step in the redesign process is to revisit the shape of the middle layer that separates the sensor from the coil. Past experiments presented in [28] showed that 5 mm-thick neoprene showed good results for normal and shear force experiments; thus, we decided to keep this material for the current iteration of the sensor. However, because the size of the coil has been significantly reduced, the neoprene shape previously presented could not be used. Instead, we decided upon a 14 mm-diameter cylindrical shape with a 5 mm hole in the center. On the previous “partial” neoprene shape [4], a volume of 2300 mm${}^{3}$ of neoprene material was used for the middle layer. The new cylindrical shape had a volume of approximately 675 mm${}^{3}$, which closely corresponds to the 1/4 size reduction of the coil. The new middle material shape is displayed in Figure 2.

**Figure 4:**
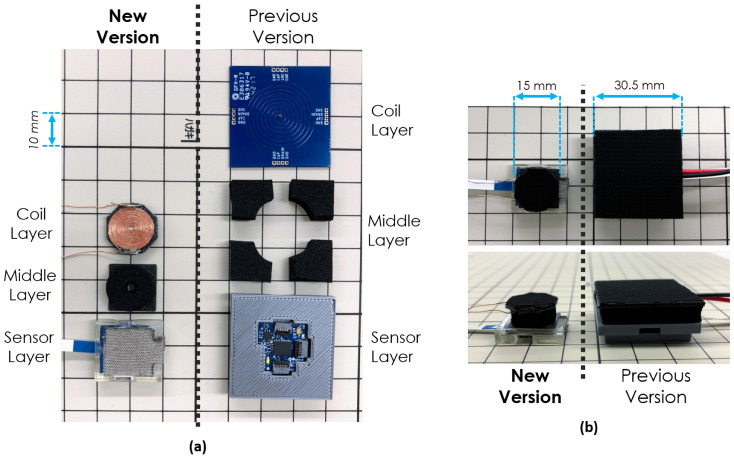
New and previous versions of the sensor module compared side by side. (**a**) Before assembly, with layer references, top view. (**b**) After assembly, top and side views.

Finally, Figure 4 shows a comparison between the new version of the sensor module and the previous iteration. Pre and post-assembly images are displayed side by side.

In addition, our new sensor module, with a size of 15×15 mm, is more compact than the skin modules of hexagonal shape, with 14 mm edges and a total footprint of approximately 28×24 mm, presented in the commendable works of [26].

### 4.3. Mitigating the High Current Requirement of the Coil Board

It becomes clear that even though the required current for the coil board has been reduced significantly, operating several sensor modules constantly and at the highest current value of 500 mA would not be practical.

Three possible ways to mitigate this problem are briefly mentioned and discussed. The options are presented in order of preference:

(a) Dormant coil strategy: Integrating the proximity capacitive sensing ability of the sensor (examined in detail further below, in Section 7), the coil of the module could be in a *dormant state* (with no current passing through it) while there is no interaction with the sensor. Then, when a threshold is reached in the proximity measurements, a trigger would *wake up* the coil to prepare it for interacting with external forces. This would reduce the energy consumption when the sensor is not in contact with any forces. A disadvantage of this method is the possibility to miss light interactions of non-conductive materials that cannot be sensed with the capacitive proximity sensor.
(b) Lower sensitivity as default setting: The sensor could be set to operate at a lower sensitivity level as the default setting, thereby utilizing less current. Moreover, because the sensor has a permanent magnet embedded into the coil board, even small values of 30% or less for the adjustable magnetic field are expected to be acceptable. Experiments would need to be conducted to settle on a practical minimum. The same disadvantage from the previous method is to be expected: that small interaction forces could be missed.
(c) Mixture of series and parallel connections: The coils could be connected in a mixture of series and parallel connections, in which case the necessary electrical power for the group of electromagnets would not change, but more practical or achievable amperage and voltage values could be reached (each new coil draws approximately 0.2 V at 500 mA). A disadvantage of this method is the added complexity in designing a network to interconnect the coils of a skin patch while following specific voltage and current targets.

Lastly, it is important to note that these options are not mutually exclusive and could be implemented in combination.

## 5. Minimizing Crosstalk between Measuring Axes

Another important aspect that we set out to address in this paper is minimizing the crosstalk between measuring axes of the magnetic sensor. The datasheet of the BMF055 SoC that we use for the sensor board does not provide precise information about the location of the magnetometer inside the integrated circuit. Without this information, in the past we decided to align the magnetic field to the center of the chip. However, by doing so, we noticed that there was significant crosstalk between axes. This was especially noticeable in the normal force experiments, where *X* and *Y* axes would increase even when only *Z* measurements should be registered. We surmise that the misalignment between the magnetic field and the center of the magnetometer is the cause of this problem.

Then, we set to probe for the location of the magnetometer inside the integrated circuit. In the experiment, we used the same XYZ table presented in the previous section, where we mounted the sensor module. On the vertical axis, we mounted a 3D-printed plastic attachment with a squared head equal in size to that of the sensor board (15×15 mm). A small permanent magnet (the same type utilized in [4]) was pasted at the center of the attachment.

**Figure 5:**
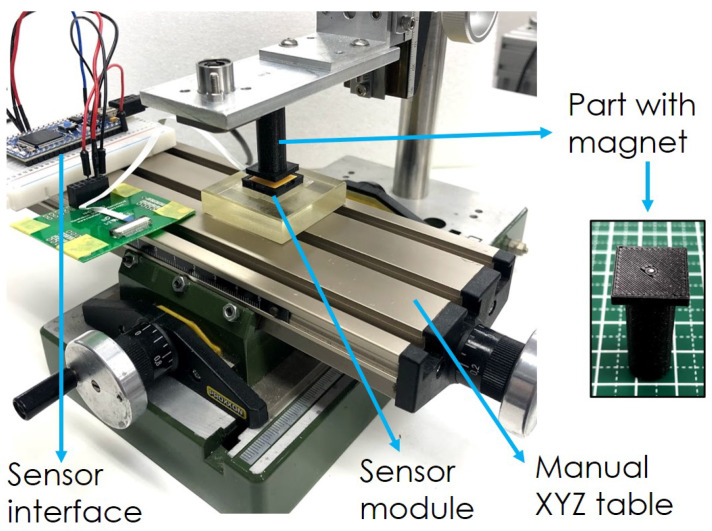
Magnetometer location experiment setup.

Initially, the attachment part with the magnet was aligned with the center of the chip. Then, the attachment part was moved up 3 mm away from the sensor in the *Z*-axis, in order to prevent sensor saturation. A picture of the setup can be found in Figure 5.

For the data acquisition, the table was manually moved in discrete points of a grid with a lattice of 0.2 mm of separation. On each point of the lattice, 15 s of magnetic measurements were recorded with the sensor board and were then averaged and assigned as the value of said lattice point. In a preliminary examination, the candidate area for the magnetometer was manually inspected, so only a necessary subset of points where the magnetometer was expected to be was measured.

**Figure 6:**
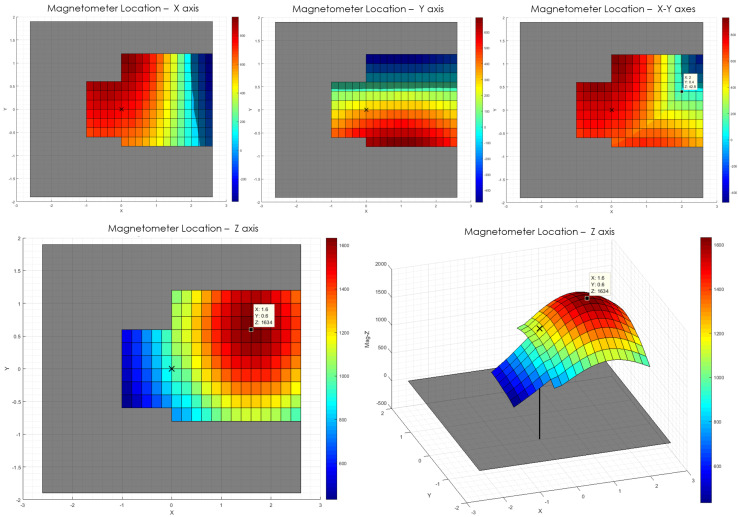
Magnetometer location experiment results. The dimensions of the BMF055 chip are displayed with a gray rectangle in the background. An “*X*” marks the center of the chip. All values are digits.

The results of this measurement are presented in Figure 6. The gray rectangle in the background represents the BMF055 SoC. Surfaces are presented for *X*, *Y*, and *Z*-axis readings (in digits). In addition, a superposition of *X* and *Y* is also presented and compared against the peak point of the *Z*-axis. The center of the magnetometer is located where the *Z*-axis measurements reach a maximum, marked with a notation. From the superposition of *X*-*Y* data, it can also be noticed that the same point where *Z* peaks, *X* and *Y* measurements have similar values (both very close to zero). This confirms the location of the magnetometer.

Therefore, the result shows that the magnetometer is likely located at (1.6,0.6), in mm, with a ±0.2 mm error, from the center of the SoC seen from the top, where the upper left corner corresponds to pin 1 of the chip.

After finding the center of the magnetometer—the main sensor within the BMF055 for our tactile sensor module—a base was designed to mount the coil in alignment with the newly found axis. To check that the crosstalk was indeed reduced, data from experiments in Section 6 were compared with readings of the sensor prior to the redesign.

**Figure 7:**
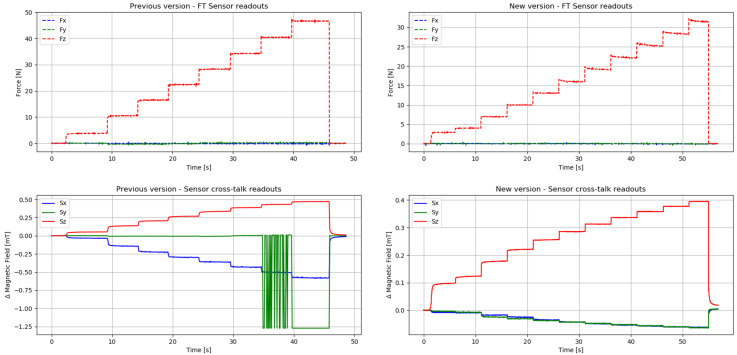
Crosstalk raw data comparison. Left: previous version of the sensor with high crosstalk values and saturation. Right: new version of the sensor with greatly reduced crosstalk values.

**Figure 8:**
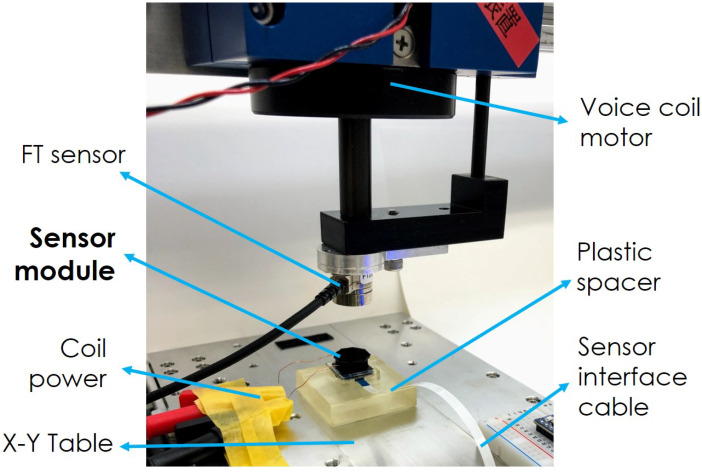
Force experiments setup.

A comparison of raw 3-axis measurement data for the previous and new designs can be seen in the graphs of Figure 7. The sensor readings correspond to normal-force-only experiments, where no shear force was applied to the module. This can be confirmed with the first row of plots, that show data from the reference force-torque sensor (FT, 6-axis force-torque sensor, Nano17 from ATI, Leawood, KS, USA) displayed in Figure 8. The maximum current values of 1500 mA and 500 mA were used on the coil for the previous and new versions, respectively.

**Figure 9:**
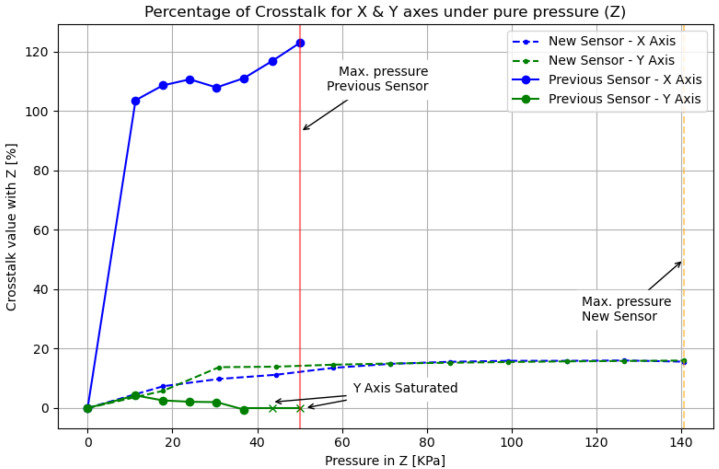
Crosstalk comparison: percentage of crosstalk from *X*-*Y* with relation to pure normal pressure (*Z*).

A direct comparison between the two sensor modules is difficult because of the significant reduction in the sensor size, the modifications of the coil and middle material characteristics, and the difference in current and forces applied to both versions. Therefore, for a more accurate comparison of crosstalk performance between both sensors, we have prepared Figure 9. The figure shows the raw data presented in Figure 7, processed to overcome the variations in sensor versions. Pure pressure (*Z*-axis only) in KPa units is used as the independent variable, and percentage of crosstalk with respect to *Z*-axis readings, as the dependent variable. Points of saturation of *Y*-axis values for the previous version and the maximum pressure values applied on both versions are annotated in the figure.

It is clear from these results that the previous version of the sensor presented a serious misalignment from the center of the magnetometer. This can be noticed by the fact that even though the main pressed axis was *Z*, the *X*-axis measured slightly larger values than on the *Z*-axis. Furthermore, towards the end of the experiment, the *Y*-axis got saturated several times. It can also be noticed that the new version of the sensor has been tested under higher pressure values and that the crosstalk remains below 20% throughout the experiment.

In the new version of the sensor, the readings from *X* and *Y* axes have been greatly reduced. The main pressed axis *Z* displays the largest measurements. Both *X* and *Y* axes still report small readings, but this is expected because the internal sensor parts for each individual axis cannot be physically located in the same position inside the SoC. In addition, the correct alignment is indicated by the fact that *X* and *Y* values produce similar readout values throughout the experiment.

Hence, crosstalk between axes has been reduced to a value that, as we verify in the next section, can be compensated by a calibration procedure applied on the sensor.

## 6. Calibration and Characterization

To determine the characteristics of the sensor, we performed several experiments. In this section, we will present details and results for sensor calibration, signal-to-noise ratio (SNR), minimum sensing value, and sensitivity adjustability curve analyses.

To collect data for the following subsections, we employed the same setup presented in [4]. The sensor was mounted horizontally on a programmable XY table (CTS150 from IKO, Antwerp, Belgium); then a 6-axis FT sensor (Nano17 from ATI, Leawood, KS, USA) and a voice coil motor (VMS05-180-LB from H2W, Santa Clarita, CA, USA) were connected in series and pressed with normal force on the sensor module.

A picture of the setup can be found in Figure 8. Plastic spacers were placed on top and below our sensor module because both the programmable XY table and the voice coil motor operate based on magnetic principles. These fields can be sensed by the geomagnetic sensor, so the plastic spacers provide distance between the experiment setup components and the sensor. The respective heights of the spacers were: 15 mm for the spacer below the sensor and 20 mm for the one on top of the sensor (the latter is not shown in Figure 8 because it blocked the view of the sensor module).

Data was acquired at 30 Hz from our sensor, a 3-fold increase from previous works, and data from the FT sensor were obtained at 100 Hz. Increasing values of normal force were applied on the sensor, reaching a maximum of 35 N. The same experiment was repeated with different current values in the coil.

### 6.1. Sensor Calibration

In order to acquire data to calibrate the sensor, two experiments were conducted using the setup described above. The first experiment was a normal force test where multiple magnitudes of force were applied to the sensor. In the second experiment, the sensor was pressed down with 7 N of normal force (to prevent slippage) and then deformed with shear force, focusing on displacing the sensor to a maximum of 2 mm from the original configuration, both on *X* and *Y* axes. The current on the coil was set to a constant value of 500 mA for these experiments.

It should be noted that the calibration procedure had to be repeated using several current values on the coil and larger forces for the shear axes in order to calibrate the sensor for the full spectrum of operation. Therefore, these results will be expanded in subsequent works.

Six linear regression models were tested to calibrate the sensor: ordinary least squares (OLS) and Huber regression methods, each with linear, quadratic, and cubic polynomial models. All regression calculations were performed using classes from the *linear_model* library of the “scikit-learn” framework [31]. Different sets of data were used for training and validation.

No filters were applied to the recorded data from the sensor or the FT sensor. However, a window of 0.5s was removed after every new force step change to remove transient responses from both sensors. Mean squared error (MSE) and coefficient of determination ($R^{2}$) values were calculated using both normal and shear force validation datasets for each of the six tested regression models. Results are presented in Table 1.

**Figure 10:**
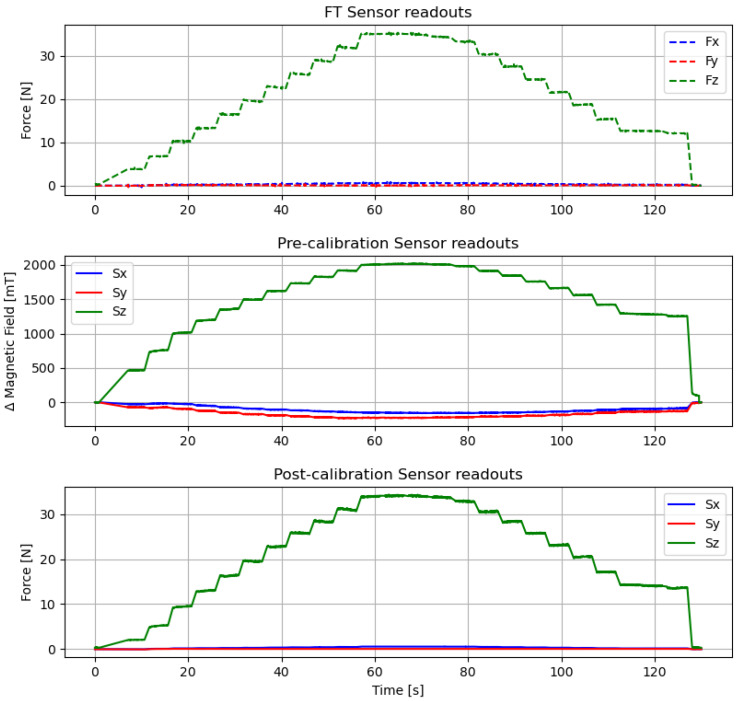
Calibration results for Huber linear regression with polynomial second-degree model. The coil was powered with 500 mA.

The method that yielded the best results was a Huber regression with a quadratic polynomial model, marked with bold font in the table. For this method, the MSE is minimum at 2.6558, and the $R^{2}$ shows a maximum value of 0.9811 for normal forces. For shear forces, MSE results remain low in all methods, while $R^{2}$ values reach levels between 0.80 and 0.90, which we consider a reasonable fit for these initial characterization tests. Figure 10 shows the processed FT sensor data, along with pre-calibration raw values and post-calibration values from the sensor module for the training dataset.

Particularly, it should be noted that the *X* and *Y* values present in the pre-calibration graph (that likely correspond to the small misalignment with the magnetic measuring axes) are flattened out by the calibration method.

**Figure 11:**
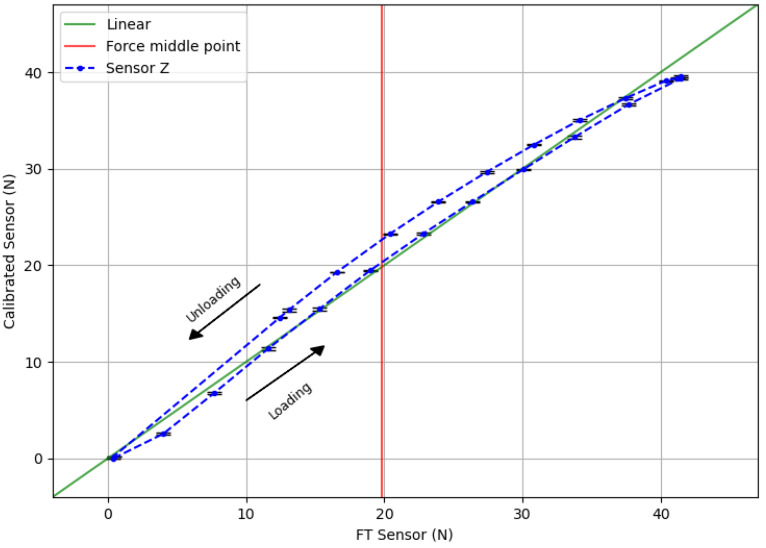
Calibrated sensor response when normal force is applied. The coil was powered with 500 mA.

**Figure 12:**
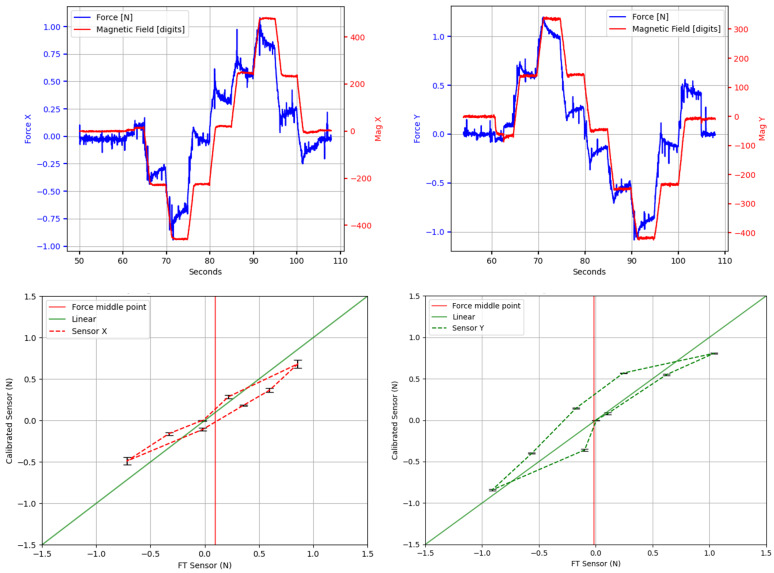
Calibrated sensor response for shear force. Top row: raw force from FT sensor and our sensor readouts for *X* and *Y*. Bottom row: Calibrated response for *X* and *Y*. Normal force of 7 N was applied on the sensor to prevent slippage. The coil was powered with 500 mA.

Figure 11 shows the calibrated sensor response for normal force values from the validation dataset. Similarly, Figure 12 shows the calibrated response of the sensor from the validation set for shear forces both in X and Y axes separately, and the raw profiles from the sensor and FT collected data. It should be noted again that the shear experiments were focusing on lateral deformation rather than maximum force values. Thus, they represent only the lower end of the shear-force-sensing spectrum. Upper limits and the influences of different current values for shear forces will be studied in detail in future works.

From these results we will calculate the hysteresis of the sensor using Equation (1).

(1) $$Hysteresis\;\%=\left|\frac{F_{mu}-F_{ml}}{F_{max}-F_{min}}\right|\times \;100\%$$

For normal force readings, $F_{ml}\left(=20.26\;N\right)$ and $F_{mu}\left(=22.60\;N\right)$ are the calibrated force values of the loading and unloading cycles, respectively, obtained using linear interpolation to the nearest neighbors. These values are taken from the midpoint force value of 19.78 N, marked with a vertical red line in the graph. $F_{min}\left(=0\;N\right)$ and $F_{max}\left(=39.55\;N\right)$ are the averaged minimum and maximum measured forces. Therefore, the calculated normal force hysteresis value of the sensor module, 5.91%, is reasonably low when compared with other magnetic sensors like [11] (with reported hysteresis for normal force close to 20%). By the same procedure, we calculate that hysteresis values for *X* and *Y*: 10.75% and 14.21%, respectively.

Considering the maximum normal force of 41.44 N used for these experiments on the validation dataset, and the new contact area after the coil size reduction (15 mm × 15 mm = 225 mm${}^{2}$), it is calculated that the sensor can handle more than 184 KPa of normal force. This represents an increase of 370% from the upper limit of the previous sensor with the PCB embedded coil, where experiments reached a maximum of ≈50 KPa (calculated from a maximum normal force of 47 N and a contact area of 30.5 mm × 30.5 mm = 930.25 mm${}^{2}$).

From the results in Figure 11 the linearity of the sensor can also be calculated. Linearity expresses, in percentage form, how much the sensor’s output value deviates from the ideal linear response. It can be calculated as:

(2) $$Nonlinearity\;\%=\frac{D_{max}}{I_{fs}}\times \;100\%$$

where, $D_{max}$ is the maximum input deviation from the linear response, and $I_{fs}$ is the maximum full-scale input. In our case, the maximum input deviation from the linear response appears at x=20.41,y=23.25 for normal force. Then, using Equation (2), the calculated linearity error of the sensor for the *Z*-axis is 7.18%. However, values for *X* and *Y* axes are less adequate, resulting in 19.47% for *X* and 19.33% for *Y*.

Smaller non-linearity percentages would be preferable for all sensing axes. In this respect, both linearity and hysteresis are highly influenced by the physical properties of the flexible material of the middle layer. Therefore, to get a more linear sensor response with a lower hysteresis value, other materials will have to be considered in future studies.

### 6.2. Signal-to-Noise Ratio

The proportion signal-to-noise ratio is a common parameter to test a sensor because it gives an idea of how noise affects the signal of the sensor and also relates to minimum sensing values. In our case, the SNR will depend on two parameters: the force applied to the sensor and the current on the coil. A way to calculate SNR is presented in [32] with the following equation:

(3) $$SNR=\frac{|\mu_{U}-\mu_{P}|}{\sigma_{u}}$$

where:

$\mu_{U}$ = not loaded sensor average value;

$\mu_{P}$ = loaded sensor average value;

$\sigma_{u}$ = not loaded sensor standard deviation value.

However, because at a fundamental level the geomagnetic sensor operates as a device that measures differences in voltage (amplitude-related quantity), we will use Equation (4) with a more convenient and comparable decibel scale.

(4) $$SNR_{dB}=20\;log_{10}\left(\frac{|\mu_{U}-\mu_{P}|}{\sigma_{u}}\right)dB$$

**Figure 13:**
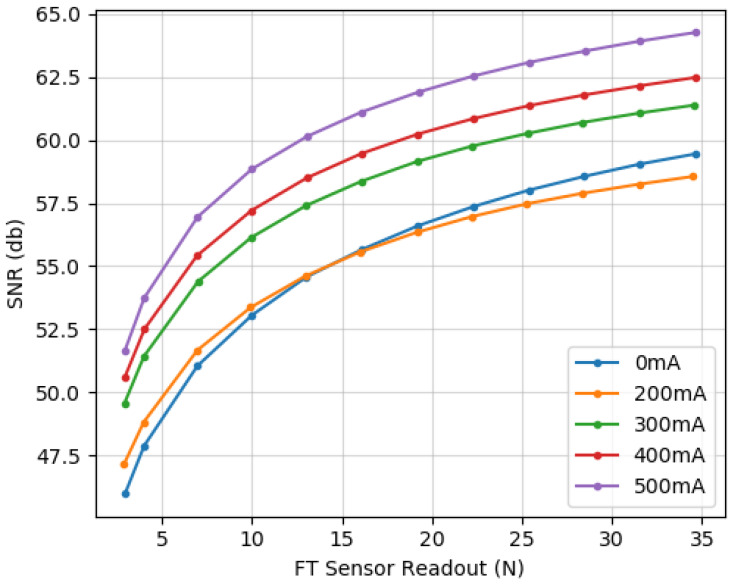
Signal-to-noise ratio (SNR) curves. Ratios change depending on the force applied. Normal force experiment readouts at five different currents on the coil.

Initially, three seconds of data of the sensor without load are considered for $\mu_{U}$ and $\sigma_{u}$. For $\mu_{P}$ values, the sensor was pressed down with increasing normal force, using the setup described above, while passing five different current values through the coil: 0, 200, 300, 400, and 500 mA. Figure 13 shows the obtained curves for SNR values.

These results verify that the sensor can reach values between 58 dB and 65 dB, which are analogous to values presented for similar magnetic sensors [33]. Moreover, we notice that the SNR is influenced more significantly by the force applied to the sensor than by the amperage on the coil. This is an important corroboration of one of the special aspects of our sensor: unlike other gain-based adjustments, we are able to increase the sensitivity of the sensor without amplifying the noise of the signal.

### 6.3. Adjustability Curves

As discussed above, unlike other sensors, our sensor module offers the possibility to adjust the sensitivity of the sensor without increasing the noise of the signal.

Therefore, if small-scale variations in force need to be discerned (for example, for interaction with delicate objects), the sensor could be set to work with high sensitivity. On the contrary, if only coarser values of force are required, the sensitivity can be set to a lower level. The possibility to adjust the sensitivity of the sensor is convenient because it broadens the sensing range without losing resolution and can be applied during operation, without the need to physically modify the sensor.

To verify this feature, data from the previously described experiments was used. The maximum normal force of 35 N was reached in increasing steps and five different current values were fed into the coil.

**Figure 14:**
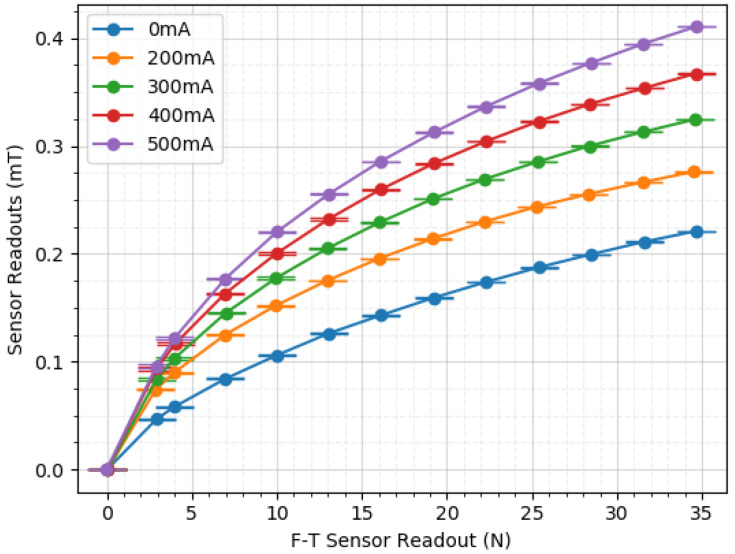
Adjustability curves. At different current values, gradients differ.

Please refer to Figure 14 for the obtained curves showing sensed magnetic field strength in the *Z*-axis (in millitesla) versus force applied (in Newtons).

From these results, it can be verified that the sensitivity of the module can indeed be adjusted. The gradient of the curves varies with the applied current value, displaying higher variation for higher currents. For example, a change of force from 4 N to 7 N produced a difference of 0.025 mT when pressed with 0 mA. However, when pressed with 500 mA, the difference grows to 0.055 mT between the same force values. Furthermore, the differences increase as the force values rise.

In previous works [29], we explained that the apparent quadratic shape of the curve is consistent with a densification area present in stress–strain curves of elastomers [34,35]. In this respect, a more linear and steeper slope would be preferable. To modify this characteristic, the polychloroprene material (neoprene) of the middle layer would need to be replaced with another material with a linear behavior. More research will be conducted in future works on the possibilities regarding this topic.

### 6.4. Minimum Detectable Force

To determine the minimum detectable force that the magnetic sensor can measure, six small non-ferromagnetic rubber objects with weights of 1, 2, 3, 4, 5, and 10 g were placed on top of the sensor. Next, ten seconds of measurements were recorded and averaged. Initial values with no weight on top of the sensor were also registered and averaged to use as a baseline value. During the experiment, the maximum current of 500 mA was applied to the coil.

**Figure 15:**
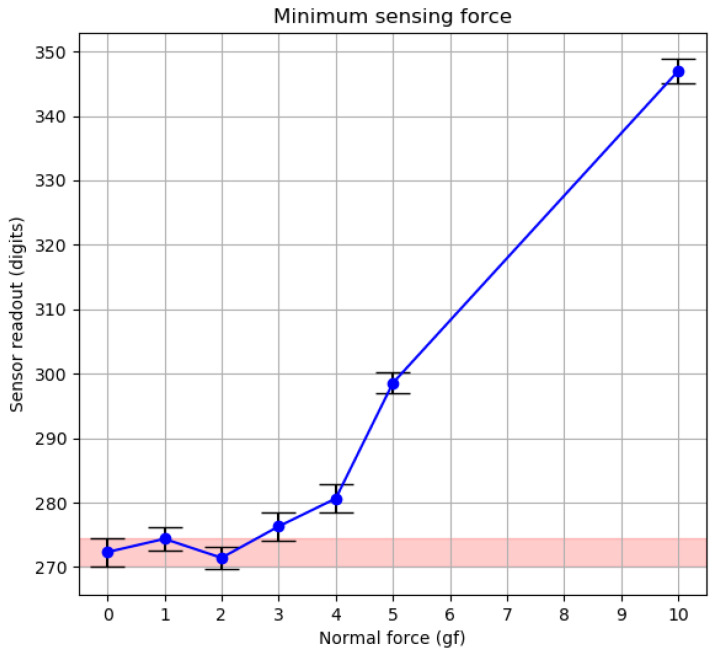
Minimum detectable normal force experiment results.

Figure 15 shows processed results. Mean and standard deviation (SD) are presented in the graph. As a visual aid, the area spanning from the SD of the baseline value has been highlighted in light red color. From the results, it can be concluded that the minimum detectable normal force of the sensor is between 3 and 4 gf (first values outside the SD area of the baseline). Notice that this value corresponds only to normal force measurements, and minimum shear force measurements will be tested at a later point.

Further details on SNR, adjustability curves, and analyses of maximum and minimum sensing comprehensive characteristics for shear forces measurements will be discussed in future works.

## 7. Multimodal Capabilities

As the final point in this letter, we offer the design, implementation, and testing of new modalities for our sensor module: proximity and normal force capacitive sensing.

By adding a layer of conductive material between the middle layer and the sensor board, the approach of a conductive object towards the module and a subsequent normal force interaction can be perceived. The location of the capacitive layer is indicated in Figure 1. This location of the capacitive pad, on top of the sensor board, was decided based on two purposes:

1. To minimize the number of cables connected to the top layer of the sensor.
2. To provide an additional sensing modality, utilizing the capacitive sensor not only as a proximity sensor but also as an additional source for normal force measurements.

Regarding the first item, a higher number of cables connecting the parts of the sensor modules that deform the upper layers could negatively influence measurements. At the moment, only cables from the coils are interconnecting the top layers of the sensor modules. Experiments to check their influence and options to mitigate negative effects will be addressed in future works.

Regarding the second item, similar to the works of [21], the sensor module is designed to operate in two modes: “Mode P” for *proximity*, and “Mode F” for *force*.

Mode P is used for proximity sensing when the sensor is not under the influence of external forces and the middle layer is not deformed. In this situation, the coil’s influence on capacitive measurements is static and the capacitive pad only would sense changes in capacitance from the approach of a conductive object (often referred to as *virtual ground*). Then, when external forces are applied to the module, the deformation produced in the middle material generates a displacement of the coil, closing the distance between the capacitive pad and the coil. At this point, the external object responsible for exerting the force on the sensor is in direct contact with the coil. Therefore, the capacitance change in this situation is related to the distance between the capacitor plates; namely, the capacitive pad and the pressing object. This effect corresponds to Mode F sensing modality. Measuring normal forces in this way has been presented by several compact tactile sensors [15,16,17,27].

Because the sensing principle of the capacitive sensor differs in nature from the magnetic and other types of sensors inside the BMF055 chip (in the sensor board), these modalities can be used as independent but complementary measurements. An added benefit from using capacitive sensing is the ability to perform pre-touch measurements even when the sensor is completely covered. While other proximity sensing technologies need holes or clear covers, our sensor modules can be entirely covered with a continuous material that does not need to be transparent. In this way, a layer of soft rubber or cloth can be placed on top of our mesh of skin sensors, adding to the compliance of the robot.

For the capacitive sensing modalities, we use the AD7147 sensor (“CapTouch Programmable Controller” from Analog Devices, Norwood, MA, USA). A patch of conductive tape (KNZ-ST50 “Shield Tape” from Kyowa Harmonet, Kyoto, Japan) is connected to a sensing pad of the AD7147, and for these initial tests, readings are processed by an Arduino Due at a rate of 250 Hz.

Currently, the capacitive sensing circuitry has been placed in a separate PCB and is not included in the sensor board. However, due to the compact size of the AD7147 chip (4×4 mm) and the few needed external electronic components, the sensor board could include the capacitive circuit on the same PCB and manage the readings with the inbuilt microcontroller.

Undesired capacitive coupling of the sensor module to the mechanical structure where it is mounted (e.g., a robot arm or body) can be minimized by the use of an active shielding mechanism offered by the AD7147 chip. In this first round of experiments, we did not use the active shield because the sensor was mounted directly on a non-conductive plastic block fixed to a wooden table.

Using a separate circuit allows conductive patches from several sensors to be connected together, providing a larger capacitive area, which in turn increases the distance sensing capabilities for the proximity measurements. Nevertheless, using a single capacitive circuit for several pads would render Mode F very impractical or impossible to implement. On the contrary, having pads discretely distributed would result in a higher sensing resolution and an improved ability for the localization of an approaching object. More advantages and disadvantages of connecting capacitive patches together versus having them as individual sensing pads have been discussed in [21]. Specific experiments for cases of use of our sensors, as well as experiments with the active shielding feature, will be presented in future works.

Experiments for Mode P and Mode F sensing modalities will be presented in the following subsections.

### 7.1. Mode P: Proximity Sensing

Mode P sensing was designed to provide the module with “pre-touch” capabilities and *anticipate* a contact event. To that end, different strategies could be applied to integrate this information in meaningful ways. For example, the dormant coil strategy presented by the end of Section 4.

As previously mentioned, capacitive proximity sensing allows the skin to be completely covered with a soft layer without the need for gaps or holes, which is an advantage over other sensing mechanisms. However, a disadvantage of this sensing modality is the fact that the approaching object needs to be made of conductive material; otherwise, capacitive readings will not detect the approach. This is a trade-off situation that we argue is more useful for real-world industrial implementation. Often the cleanliness of industrial environments is insufficient, so any aperture in sensor covers (or sensors without covers) could get blocked due to dust or grease, or accelerate breakdown from normal usage.

**Figure 16:**
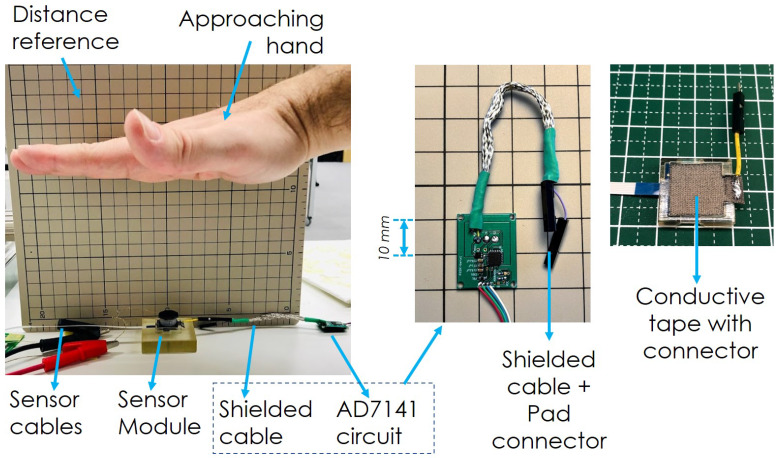
Test setup for “pre-touch” sensing. Hand approaches from the top, closing the distance to the sensor. Details on capacitive sensor PCB and conductive tape with connector from sensor.

In order to test this new pre-touch modality, we horizontally placed the fully connected sensor on a tabletop and the hand of a human test subject approached from the top, closing the distance between the hand and the sensor. A picture of the test setup can be found in Figure 16. For the hand approach experiment, a collection of 10 s of data was recorded for each distance point to minimize the small movements of the hand in the mean calculation. Readout measurements from the AD7147 sensor include a 5-s record at the beginning of the experiment that is averaged and used as a baseline for subsequent values; therefore, all reported readouts are offsets from this initial baseline.

**Figure 17:**
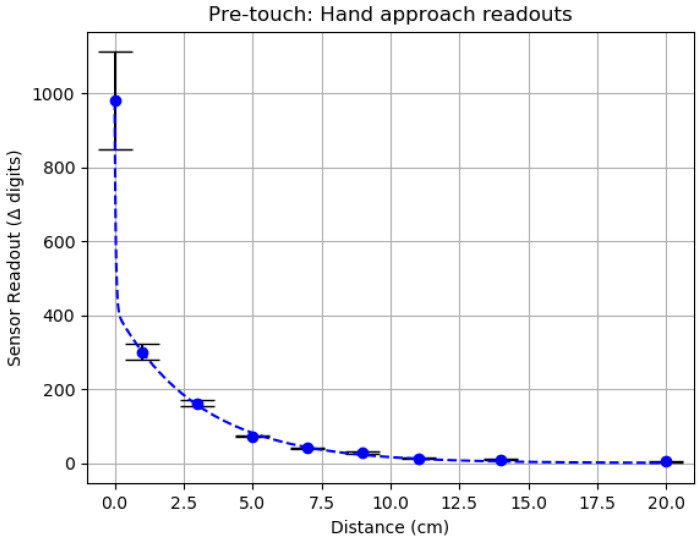
Capacitive proximity sensing: “pre-touch” experiment results.

Results of the experiments are presented in Figure 17, alongside a table with the values (Table 2).

The pre-touch results show that the approach of the hand generated an expected exponential reply on the capacitive sensor. A fitting curve was calculated using the *Optimization* library from *SciPy* [36] with a nonlinear least-squares solver and a two-terms exponential model. The resulting equation is the following: $y=a\;e^{\;b\;x}+c\;e^{\;d\;x}$ where a=417.068, b=−0.327, c=563.02, and d=−25.315. Furthermore, it can be noticed that the SD values increase as the distance from the sensor decreases. We propose that this is because of small vibrations of the hand during the experiment, that correspond to the larger fluctuations in sensing values when the hand is near the sensing pad. In the case of this experiment, the approaching object seems to be detectable with some level of confidence from a distance of around 10 cm when the reported sensor values are close to 30 units over the baseline.

In an extended skin patch with several sensor modules interconnected, the matrix of coils would partially cover the capacitive pads, possibly exerting some influence on the sensing limits or the practical use of Mode P. Such situations will be analyzed in future works.

### 7.2. Mode F: Normal Force Sensing

Mode F was confirmed by an experiment devised to check the behavior of the capacitive sensing modality when force is applied onto the sensor. To assert that the Mode F results would not be influenced by the material of the object exerting the force, two sets of objects were used: conductive and non-conductive. In the first case, five conductive objects from a weight calibration set made from grade 45 steel were placed on the sensor (values: 5, 10, 20, 50, and 100 g). As for non-conductive objects, different weighted quantities of uncooked dry rice were placed in a plastic container made from polyethylene, which was then placed on the sensor (values: 1, 2, 3, 4, 5, 10, 20, 50, and 100 g).

**Figure 18:**
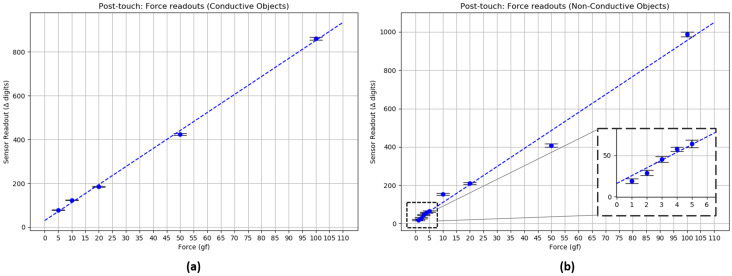
Capacitive normal force sensing: “post-touch” experiment results. (**a**) Conductive objects (steel). (**b**) Non-Conductive objects (dry uncooked rice), with inlay details for small weight values of 1–5 gf.

The weight values employed were purposefully selected to check if the limits of the lower end of the magnetic force sensor could be compensated, especially in the case of non-conductive materials, which could represent a weaker secondary capacitor plate. All objects have been weighed before the experiment with a 0.01 g resolution scale. Eight seconds of data were registered for each object, mean and SD values are reported on the graphs. During the experiments for both sets of objects, the sensor module was fully connected, with the coil powered to the maximum of 500 mA and sensor board readings broadcasting continuously. Figure 18 shows the results of these experiments.

Interestingly, both sets of objects (conductive and non-conductive) seem to behave similarly, in a linear fashion, for normal force measurements. This result might be the outcome of a convolution between the capacitive measurement and the quadratic deformation profile of the middle material. This hypothesis, along with others, will be verified at a later point.

Linear models fit both sets of data with the following equation format: y=ax+b where a=8.221 and b=30.051 for the conductive set of objects, and a=9.393 and b=15.927 for the non-conductive set.

From Figure 17 and Figure 18, it can be noticed that Mode P and Mode F feature slopes with different characteristics. This is one aspect that could help discern between modes.

Therefore, Mode F shows promising results that could provide additional normal force measurements, especially for low ranges of force (from 1 gf ) that the magnetic force sensor cannot reach. Further research will be conducted on detecting upper limits for these modalities, strategies to integrate and complement the normal magnetic-based force readings, and to provide a more in-depth analysis of the capacitive modes along with their characterization.

## 8. Conclusions and Future Works

As tactile sensing can offer additional and critical information, helping robots to safely interact with people and their environments, we have further developed a multimodal, adjustable sensitivity skin sensor module. The footprint of the sensor has been reduced by 1/4 and the coil layer and middle layer were redesigned, implemented, and tested to confirm that the characteristics of the sensor remained appropriate to prior requirements.

The problem of high crosstalk between axes has been reduced in a significant amount by detecting the location of the magnetometer within the BMF055 chip and redesigning the sensor mounting structure to align the magnetic field of the coil board with the magnetometer. The reduction in crosstalk signals has been verified with experiments. Furthermore, the small percentage of misalignment that remained could be successfully compensated during the calibration process.

Six calibration methods were examined and a Huber regression with a second-degree polynomial model was selected. For normal force, the hysteresis was calculated to be 5.91%, while SNR values range from 45 dB to 64 dB, both values comparable to characteristics of similar magnetic sensors.

Minimum detectable force experiment results showed that the lower limit the magnetic sensor can measure is ≈4 gf (with the maximum current of 500 mA in the coil). Meanwhile, the upper limit was tested with 41 N of normal force (4.18K gf or 184 KPa considering the new contact area). This represents an increase of 370% higher value than the one used for the previous iteration.

Sensitivity adjustability curves were presented to demonstrate that the sensitivity of the sensor can be adjusted by modifying the current that is passing through the coil. SNR curves support the fact that the noise ratio stays low throughout different forces applied to the sensor and different current values on the coil.

Lastly, two new modalities for proximity (Mode P) and normal force sensing (Mode F) were implemented employing a capacitive sensor circuit. Experiments indicate that Mode P could detect the approach of a human hand from a distance of 10 cm, and Mode F could be used for sensing lower normal force values that the magnetic sensor cannot reach, possibly as low as 1 g, while providing additional useful data for normal force measurements.

Considerations between integrating the capacitive circuit in a single sensor module against multiple modules remain for future works. Additionally, in this iteration, shear force experiments were performed with focus only on the lower end of the sensing spectrum, so further experimentation is required for a detailed characterization under the influence of higher shear forces.

Multi-module connections and strategies to process information in a mesh structure should also be studied in the future.

## Figures and Tables

**Table 1:** Mean squared error (MSE) and coefficient of determination ($R^{2}$) values for regression models. Normal and shear forces.

Regression Model	Normal		Shear			
	MSE (Z)	$R^{2}$ (Z)	MSE (X)	$R^{2}$ (X)	MSE (Y)	$R^{2}$ (Y)
OLS + Linear Model	32.5288	0.7681	0.0299	0.8154	0.0366	0.8651
OLS + Quadratic Model	5.4828	0.9609	0.0190	0.8831	0.0343	0.8733
OLS + Cubic Model	59.3913	0.5766	0.0171	0.8944	0.0307	0.8867
Huber + Linear Model	44.0833	0.6857	0.0304	0.8123	0.0383	0.8586
**Huber + Quadratic Model**	**2.6558**	**0.9811**	**0.0255**	**0.8428**	**0.0398**	**0.8531**
Huber + Cubic Model	24.0075	0.8288	0.0193	0.8808	0.0324	0.8803

**Table 2:** “Pre-touch” experiment values.

Distance (cm)	Sensor Readouts (Δ Digits)
20	4.25±1.191
14	8.976±1.766
11	13.200±1.660
9	28.392±1.755
7	40.014±2.081
5	73.036±2.584
3	162.013±7.659
1	299.989±21.367
0 (contact)	980.087±131.804

## Abbreviations

The following abbreviations are used in this manuscript:

MTS	Manipulation tactile sensors
ETS	Extended tactile sensors
PCB	Printed circuit board
AWG	American wire gauge
SoC	System on chip
FT	Force-torque
SNR	Signal-to-noise ratio
OLS	Ordinary least squares
$R^{2}$	Coefficient of determination
MSE	Mean squared error
SD	Standard deviation
